# Liver Fibrosis and Risk of Incident Dementia in the General Population: Systematic Review With Meta‐Analysis

**DOI:** 10.1002/hsr2.71530

**Published:** 2025-11-17

**Authors:** Mohamad Jamalinia, Fatemeh Zare, Amedeo Lonardo

**Affiliations:** ^1^ Gastroenterohepatology Research Center Shiraz University of Medical Sciences Shiraz Iran; ^2^ Department of Internal Medicine Azienda Ospedaliero‐Universitaria di Modena (−2023) Modena Italy

**Keywords:** chronic liver disease, dementia, liver‐brain axis, MASLD, NAFLD, sex differences

## Abstract

**Background and Aims:**

The relationship between liver fibrosis and the risk of developing dementia remains unclear, with studies yielding inconsistent results. This systematic review and meta‐analysis seek to synthesize the available evidence.

**Methods:**

We systematically searched PubMed, Scopus, Embase, and Web of Science from their respective inception through October 2024 to identify observational studies diagnosing liver fibrosis non‐invasively or via histology. The primary outcome was new‐onset dementia. Risk of bias was evaluated using the Newcastle‐Ottawa Scale (NOS), and pooled estimates of hazard ratios (HRs) with 95% confidence intervals (CIs) were calculated using a random‐effects model.

**Results:**

Eight longitudinal cohorts, including 1,115,759 middle‐aged individuals (31,129 with liver fibrosis at baseline), identified 29,923 new dementia cases over a mean follow‐up of 14 years. Liver fibrosis exhibited a 32% increased risk of developing all‐cause dementia (pooled HR: 1.32, 95% CI: 1.08–1.61; *I*² = 76.06%). Dementia risk increased with fibrosis severity: HR 1.06 (95% CI: 0.67–1.68) in ≥F2, HR 1.32 (95% CI: 1.06–1.64) in ≥F3, and HR 1.69 (95% CI: 1.01–2.83) in F4. Geographically, the risk appeared higher in Western than Eastern countries. Women had a greater risk, and vascular dementia was more strongly associated with fibrosis than Alzheimer's disease. Sensitivity analyses confirmed the robustness of the findings, and no publication bias was observed.

**Conclusion:**

Liver fibrosis is linked to a 32% increased long‐term dementia risk, independent of common demographic, social, anthropometric, and cardiometabolic factors. Fibrosis severity further increases this risk. Based on our findings, healthcare professionals should recognize the moderately increased risk of developing dementia in individuals with liver fibrosis and perform close surveillance of these patients to enable early detection and timely intervention.

## 1 Introduction

Dementia is a progressive decline in cognitive function that impairs independent living and represents one of the most pressing healthcare and public health challenges among ageing populations [1, 2]. Affecting over 55 million people worldwide, dementia is the seventh leading cause of death, significantly contributing to disability, with an estimated global cost of $1.3 trillion in 2019 [3]. Due to population aging, its prevalence is expected to double every 20 years, reaching 78 million in 2030 and 139 million in 2050 [4]. There are two major types of dementia, Alzheimer's disease (AD) and vascular dementia (VD), with women being at higher risk for AD [5] and experiencing greater dementia‐related disability‐adjusted life years (DALYs) and mortality [3]. Given the limited treatment options and the heterogeneity of underlying pathomechanisms, identifying modifiable risk factors for cognitive decline is crucial for effective prevention [6, 7]

Among medical comorbidities contributing to dementia, studies have expanded the scope of the liver‐brain axis to encompass the entire spectrum of chronic liver disease (CLD) [8]. Liver fibrosis, a hallmark of CLD, is characterized by excessive extracellular matrix deposition resulting from repeated inflammation and tissue repair processes, irrespective of the underlying etiology [9]. Advanced fibrosis alters liver architecture and function, leading to portal hypertension, hepatic insufficiency, hepatocellular carcinoma, and, in some cases, the need for liver transplantation [10].

Recent studies have proposed liver fibrosis as a potential risk factor for incident dementia [7, 11], possibly due to increased intracerebral pro‐inflammatory cytokines, insulin resistance, oxidative stress, free fatty acids overflow, impaired mitochondrial homeostasis, and cerebrovascular dysfunction associated with the prothrombotic state in CLD [12, 13]. Achieving sustained virological response (SVR) after antiviral therapy for hepatitis C virus (HCV) has also been associated with a significantly lower risk of developing dementia compared to untreated individuals [14].

However, studies published so far have yielded conflicting findings, with other publications not confirming a nexus between liver fibrosis and dementia [15, 16]. To address these uncertainties, we conducted a systematic review with meta‐analysis focusing on incident dementia, its subtypes, and sex‐specific analysis of data.

## 2 Methods

### 2.1 Protocol and Search Strategy

This systematic review was conducted in accordance with the Preferred Reporting Items for Systematic Reviews and Meta‐Analyses (PRISMA) framework [17]. Given that the included studies were observational, we also adhered to the Meta‐analysis of Observational Studies in Epidemiology (MOOSE) criteria for conducting and reporting meta‐analyses [18]. A comprehensive search across four major electronic databases: PubMed, Embase, Scopus, and Web of Science, from their inception up to October 22, 2024. The aim was to identify observational studies exploring the risk of new‐onset dementia in adults (≥ 18 years) with and without liver fibrosis. A combination of Medical Subject Headings (MeSH) terms, Emtree terms, and relevant keywords was used, with the complete search strategies outlined in Supporting Information S1: Tables S1 and S2.

Two independent reviewers (M.J. and F.Z.) initially screened the titles and abstracts of all identified studies based on predefined inclusion and exclusion criteria. Full‐text articles were then retrieved and assessed for eligibility by the same two reviewers, with any disagreements resolved through consultation with the third reviewer (A.L.). Additionally, we manually searched the reference lists of selected articles to identify any relevant studies not captured in the electronic search.

The review protocol was prospectively registered in the International Prospective Register of Systematic Reviews (PROSPERO) under registration number CRD42024604692. As this study involved secondary analysis of previously published studies, no additional ethical approval was necessary. All original studies included in the review had relevant ethical approvals and, where applicable, obtained informed consent from participants.

### 2.2 Eligibility Criteria

To be included in this meta‐analysis, studies had to meet the following criteria [1]: prospective or retrospective cohort studies investigating the link between liver fibrosis and the risk of developing dementia [2]; provision of hazard ratios (HRs) with 95% confidence intervals (CIs) as measures of association; and [3] assessment of liver fibrosis using validated biomarkers, fibrosis scoring systems (e.g., FIB‐4, NFS), international classification of diseases (ICD)‐coded diagnoses, imaging techniques, or histological evaluation. The analysis encompassed adult participants across all demographic groups, with no exclusions based on race or ethnicity.

Studies were excluded from our meta‐analysis based on any of the following criteria [1]: non‐eligible study designs, including conference abstracts, case reports, reviews, commentaries, or cross‐sectional studies [2]; cohort studies lacking HRs with 95% CIs for the specified outcome [3]; cohort studies that exclusively examined individuals with liver fibrosis or cirrhosis without a comparison group of those without these conditions; or [4] cohort studies restricted to participants with pre‐existing dementia.

### 2.3 Data Extraction and Quality Assessments

Two reviewers (M.J. and F.Z.) independently extracted pertinent data from eligible studies and recorded it in Microsoft Excel. The extracted data encompassed the author names, publication years, study design, country of origin, population characteristics, methods used to diagnose fibrosis, follow‐up length, key outcomes, and variables controlled for in multivariable analyses, such as matching or confounders. In cases where multiple publications were based on the same cohort, we included the study with the most specific cutoff for liver fibrosis in the overall analysis. Other studies from the same cohort were included in subgroup analyses if they employed different methods for specific subgroups.

The Newcastle–Ottawa Scale (NOS) was used independently by the same two reviewers (M.J. and F.Z.) to evaluate the quality of each included study. Discrepancies were resolved through discussion and, when necessary, consultation with a third reviewer (A.L.). The NOS employs a star‐based rating system to evaluate studies across three key areas: individual selection (maximum of four stars), comparability of groups in the study (up to two stars), and outcome assessment (up to three stars). Studies awarded 9 stars were deemed to have a low risk of bias, 7 or 8 a moderate risk, and 6 or fewer a high risk. Reviewer assessments for these domains were documented using the risk of bias tool available in Review Manager software [19]. Additionally, NOS scores were weighed by the relative significance of each study, as illustrated in Figure 2, to account for the potential bias introduced by studies with a higher risk of bias (lower NOS scores) in the overall outcome.

### 2.4 Data Synthesis and Analysis

Statistical analysis was performed using the “metan” and “metabias” packages in Stata 16 (StataCorp LLC, College Station, TX). The DerSimonian‐Laird random‐effects model was employed to estimate the summary effect size, using the natural logarithm of the hazard ratio (HR) as the effect measure to combine the study outcomes. The results are presented as pooled HRs with 95% confidence intervals (CIs). For studies presenting multiple HRs with different levels of covariate adjustment, we selected and pooled the HRs that accounted for the most extensive set of confounders, applying the inverse variance methods. If studies reported multiple hazard ratios for different ranges of liver fibrosis, we excluded results from patients with inconclusive fibrosis diagnoses, as these correspond to highly heterogeneous populations with significant fibrosis variability. Heterogeneity between studies was assessed using the Q statistic and *I*². The interpretation of *I*² values was as follows: 0%–25% indicated minimal heterogeneity, 26%–50% moderate heterogeneity, 51%–75% substantial heterogeneity, and 76%–100% considerable heterogeneity [20]. A funnel plot and Egger's regression test were conducted to evaluate publication bias.

As prespecified in our registered protocol, we performed subgroup analyses based on fibrosis severity, classified according to the METAVIR scoring system into three groups: significant fibrosis (stage F2 or higher), advanced fibrosis (stage F3 or higher), and cirrhosis (stage F4) [21]. Significant fibrosis (≥ F2) was defined via liver biopsy or liver stiffness measurement (LSM) > 8 kPa [22]. Advanced fibrosis (≥ F3) was defined as FIB‐4 > 2.67 or NAFLD fibrosis score (NFS) > 0.676 [23]. Cirrhosis (F4) was identified using liver biopsy findings or ICD codes for cirrhosis.

After the data were gathered, we performed additional analyses to identify possible sources of heterogeneity and evaluate the stability of the observed associations. These included subgroup analyses based on study region, follow‐up length, methods of dementia diagnosis, fibrosis assessment techniques, study quality, and the extent of covariate adjustment. Three sensitivity analyses were also performed [1]: including only studies with the highest level of covariate adjustment [2], limiting to studies with low risk of bias (NOS score of 9), and [3] a leave‐one‐out analysis, systematically excluding each study to assess its influence on the overall estimate. In addition, we performed cause‐specific analyses by pooling effect sizes separately for vascular dementia and Alzheimer's disease, and sex‐specific analyses by pooling effect sizes separately for men and women. All statistical analyses were two‐sided, and *p* values < 0.05 were considered statistically significant.

## 3 Results

### 3.1 Characteristics of Eligible Cohorts

**Figure 1:**
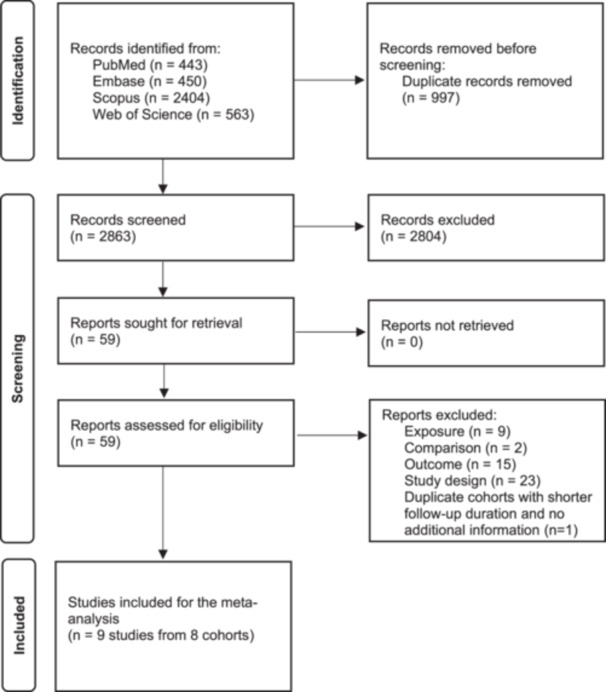
PRISMA flow diagram outlining the study screening and selection process.

We systematically searched the literature and identified 2913 studies after removing duplicates. Of these, 2854 studies were excluded based on title and abstract screening. We reviewed the full text of the remaining 59 potentially relevant articles and ultimately included 9 studies [8, 11, 14, 15, 16, 24, 25, 26, 27] from 8 cohorts included in our analysis. A detailed flowchart of the study screening and selection process is provided in Figure 1.

The main characteristics of the eight eligible cohorts are summarized in Table 1. Most cohorts recruited participants from general populations or large health examination programs. Geographically, four cohorts were conducted in European countries (Italy, Sweden, the Netherlands, and the United Kingdom), two in the United States, and two in Asia (Taiwan and Israel).

**Table 1:** Eligible cohort studies examining the association between liver fibrosis and risk of developing incident dementia (*n* = 9).

Author, year	Study characteristics	Fibrosis diagnosis (fibrosis patients, *n*)	Dementia outcome (incident dementia events, *n*)	Main findings	Covariate adjustments	NOS
Solfrizzi et al., 2020	Population‐based cohort study (Italian Longitudinal Study on Aging, Italy): 1061 older adults (mean age: 72 years, 44% women). Excluded prevalent dementia, Parkinson's disease, stroke, viral hepatitis, and patients not screened for frailty syndrome. Mean follow‐up: 8 years.	NFS (≥ F3, *n* = 141)	Incident all‐cause dementia diagnosed using DSM‐III‐R, NINCDS‐ADRDA (Alzheimer's disease), and ICD‐10 (vascular dementia). (*n* = 90)	Compared to patients with lower fibrosis scores (F0‐F2), advanced liver fibrosis (F3‐F4 NFS) did not independently predict dementia risk (aHR: 0.91, 95% CI: 0.44–1.88).	Age, sex, metabolic syndrome, alcohol, Smoking, education, Charlson comorbidity index, MMSE score at baseline, serum albumin, apolipoprotein B/A1 ratio.	8
Wen et al., 2021	Nationwide population‐based cohort study (Taiwan NHIRD, Taiwan): 10,578 AMD patients matched 1:1 with controls (50.8% men, mean age: 70.5 years). Excluded individuals with pre‐existing Alzheimer's disease. Follow‐up: 5.68 years.	ICD‐9 (F4, *n* = 7357)	Alzheimer's disease diagnosed using ICD‐9 codes. (*n* = 549)	Compared to patients without cirrhosis, cirrhosis did not predict Alzheimer's disease risk (HR: 1.01, 95% CI: 0.85–1.21).	None	7
Shang et al., 2021	Retrospective cohort study (Sweden): 656 biopsy‐confirmed NAFLD patients (mean age: 48 years, 37% women), matched 1:10 with 6436 controls. Excluded prevalent dementia, excessive alcohol use, and non‐NAFLD steatosis. Mean follow‐up: 19.7 years.	Biopsy (≥ F2, *n* = 227; F4, *n* = 20)	All‐cause dementia diagnosed using ICD 8‐10 codes. (*n* = 340)	Compared to the matched control group, neither NAFLD with significant fibrosis ( ≥ F2, aHR: 1.04, 95% CI: 0.52–2.11) nor cirrhosis (F4, aHR: 1.72, 95% CI: 0.35–8.46) was significantly associated with an increased risk of dementia. Similarly, in the cause‐specific analysis for Alzheimer's disease, no significant association was observed for either significant fibrosis ( ≥ F2, aHR: 0.99, 95% CI: 0.41–2.36) or cirrhosis (F4, aHR: 2.43, 95% CI: 0.45–12.90).	Age, sex, and cardiovascular disease.	7
Parikh et al., 2022	Retrospective cohort study (UK Biobank, United Kingdom): 455,226 participants (mean age: 56 years, 54% women). Excluded participants with prevalent dementia, acute hepatitis, or severe thrombocytopenia. Median follow‐up: 9 years.	FIB‐4 > 2.67 (≥ F3, *n* = 9895).	Incident all‐cause dementia diagnosed using validated ICD‐9/ICD‐10 algorithms. (*n* = 2223)	Compared to patients with FIB‐4 ≤ 2.67, those with FIB‐4 > 2.67 had higher all‐cause dementia risk (aHR: 1.52, 95% CI: 1.22–1.90). Significant for vascular dementia (HR: 1.64, 95% CI: 1.03–2.60), but not Alzheimer's disease (HR: 1.31, 95% CI: 0.89–1.93). Sensitivity analyses confirmed the associations.	Age, sex, race/ethnicity, educational attainment, socioeconomic deprivation, hypertension, diabetes, dyslipidemia, BMI, metabolic syndrome, smoking, alcohol use.	9
Xiao et al., 2022	Prospective cohort study (Rotterdam Study, Netherlands): 3300 participants with liver stiffness data (mean age: 67 years, 57% women). Excluded secondary steatosis, viral hepatitis, prevalent dementia, and significant alcohol use. Median follow‐up: 5.6 years.	Elastography, liver stiffness ≥ 8.0 kPa (≥ F2, *n* = 192).	All‐cause dementia diagnosed via MMSE, GMS, CAMDEX, and confirmed using DSM‐III‐R criteria. (*n* = 127)	Compared to patients with liver stiffness < 8.0 kPa, those with liver stiffness ≥ 8.0 kPa were not significantly associated with dementia risk (aHR: 1.07, 95% CI: 0.58–1.99).	Age, sex, education, APOE ε4, alcohol, smoking, cholesterol, stroke, hypertension, diabetes, BMI.	9
Tao et al., 2023	Retrospective/prospective cohort (CHeCS, United States): 17,485 HCV patients (mean age: 50 years, 40% women). Excluded prevalent dementia, liver transplant, and HBV co‐infection. Mean follow‐up: 6.7 years	ICD‐9 (F4, *n* = 3762)	All‐cause dementia diagnosed using ICD‐9 codes. (*n* = 342)	Compared to patients without cirrhosis, cirrhosis doubled dementia risk (aHR: 2.05, 95% CI: 1.60–2.61). Additionally, SVR had a significantly decreased risk of dementia compared to untreated patients (aHR: 0.32, 95% CI: 0.22−0.46)	Age, sex, race, BMI, smoking, diabetes, insurance type, HCV treatment, and Charlson comorbidity index.	8
Lu et al., 2023	Prospective cohort (ARIC Study, United States): 8972 midlife participants (mean age: 57 years, 55% women). Excluded prevalent stroke/dementia, significant alcohol consumption, and clinical liver disease. Median follow‐up: 24.5 years.	FIB‐4 > 2.67 (≥ F3, *n* = 214)	All‐cause dementia diagnosed using neuropsychological assessments, informant reports, and medical records. (*n* = 1789)	Compared to patients without NAFLD and with low fibrosis risk, NAFLD with advanced fibrosis was associated with increased dementia risk (aHR: 1.94, 95% CI: 1.01–3.71).	Age, sex, race, education, APOE ε4 genotype, alcohol use, eGFR, BMI, blood pressure, HDL, total cholesterol, hypertension, and diabetes.	9
Yuan et al., 2024	Prospective cohort (UK Biobank, United Kingdom): 458,181 participants (mean age: 56 years, 55% women). Excluded prevalent dementia and 14 digestive system diseases. Median follow‐up: 12.4 years.	ICD‐9/ICD‐10 (F4, *n* = 871)	All‐cause dementia diagnosed using ICD‐9/ICD‐10 codes. (*n* = 6415)	Compared to patients without digestive system diseases, cirrhosis significantly increased the risk of dementia (aHR: 2.31, 95% CI: 1.98–2.70). Cirrhosis was associated with both vascular dementia (aHR: 2.20, 95% CI: 1.55–3.13) and Alzheimer's disease (aHR: 1.88, 95% CI: 1.43–2.47). Additionally, this risk was higher in women (aHR: 3.23, 95% CI: 2.10–4.97) than in men (aHR: 1.45, 95% CI: 0.82–2.56).	Age, sex, Townsend deprivation index, education, BMI, physical activity, diet, smoking, alcohol, hypertension, stroke, family history of dementia, depression, and polygenic risk score.	9
Weinstein et al., 2024	Retrospective cohort study (Clalit Health Services, Israel): 826,578 participants (mean age: 55 years, 56% women). Excluded prevalent dementia, excessive alcohol use, and chronic liver/gastrointestinal diseases. Median follow‐up: 17 years.	FIB‐4 ≥ 2.67 (≥ F3, *n* = 8897)	Incident all‐cause dementia assessed using ICD‐9/ICD‐10 codes, hospital discharge data, and anti‐dementia medication records.(*n* = 23,817)	Compared to patients with FIB‐4 < 1.3, advanced fibrosis (FIB‐4 ≥ 2.67) increased dementia risk (aHR: 1.18, 95% CI: 1.10–1.27). Results were robust in sensitivity analyses. Additionally, this risk was slightly higher in women (aHR: 1.22, 95% CI: 1.10–1.35) than in men (aHR: 1.17, 95% CI: 1.06–1.29).	Age, sex, ethnicity, peripherality, smoking, obesity, diabetes, hypertension, hyperlipidemia, ischemic heart disease, carotid artery stenosis, history of stroke, anxiety, depression, Parkinson's disease.	9

Abbreviations: AD8, eight‐item dementia screening interview; AMD, age‐related macular degeneration; APOE ε4, apolipoprotein E ε4 genotype; ARIC, atherosclerosis risk in communities; BMI, body mass index; CAMDEX, Cambridge examination for mental disorders of the elderly; CDR, clinical dementia rating; CHeCS, chronic hepatitis cohort study; DSM‐III‐R, diagnostic and statistical manual of mental disorders‐third edition, revised; eGFR, estimated glomerular filtration rate; FAQ, functional activities questionnaire; FIB‐4, fibrosis‐4 index; GMS, geriatric mental state examination; HBV, hepatitis B virus; HCV, hepatitis C virus; ICD, International Classification of Diseases; MMSE, mini‐mental state examination; NAFLD, nonalcoholic fatty liver disease; NFS, nonalcoholic fatty liver disease fibrosis score; NINCDS–ADRDA, National institute of neurological and communicative disorders and Stroke–Alzheimer's disease and related disorders association; SIS, six‐item screener; SVR, sustained virological response; TICSm, telephone interview for cognitive status–modified; UK Biobank, United Kingdom Biobank.

Regarding fibrosis diagnosis methods, one cohort utilized liver biopsy, one used imaging modalities, and three used blood biomarkers/scores (FIB‐4 and NFS). Two cohorts relied on ICD codes for fibrosis diagnosis. Additionally, studies from the UK Biobank employed different methods, with one using FIB‐4 and another using ICD codes. Dementia diagnoses were also based on ICD codes in six studies, while the remaining two used validated neuropsychological assessments and medical records.

The cohorts assessed different fibrosis levels. Two cohorts provided data on significant liver fibrosis (≥ F2), four on advanced liver fibrosis (≥ F3), and four on cirrhosis (F4). Among the included studies, five cohorts accounted for the most comprehensive set of confounders, including age, sex, body composition metrics, smoking habit, alcohol consumption, blood pressure or hypertension, lipid levels or dyslipidemia, and diabetes status or fasting glucose levels. The remaining three studies applied adjustments for a subset of these factors.

Dementia was defined as all‐cause dementia in seven cohorts. The UK Biobank cohorts additionally reported separate outcomes for AD and VD. One study reported outcomes for both all‐cause dementia and AD, while another study reported only AD as the outcome of interest. Lastly, two cohorts provided sex‐specific outcomes for both males and females.

### 3.2 Publication Bias and Quality Assessments

The Egger regression test yielded a *p* value of 0.79, indicating publication bias was unlikely in our analysis. Similarly, the funnel plot analysis (Supporting Information S1: Figure S1) showed no apparent asymmetry, further supporting the absence of substantial publication bias. Therefore, publication bias is unlikely to have a meaningful impact on the findings of this systematic review.

As summarized in Table 1 and Supporting Information S1: Table S3, five studies received nine stars on the NOS, indicating a low risk of bias, while three studies received eight or fewer stars, signifying an overall moderate to low risk of bias. Risk of bias in our meta‐analysis was further visualized using the Cochrane Collaboration tool, as shown in Supporting Information S1: Figures S3 and S4. The pooled outcome had a mean weighted NOS score of 8.28, indicating a generally moderate to low risk of bias across the included studies.

### 3.3 Liver Fibrosis and Risk of Incident Dementia

**Figure 2:**
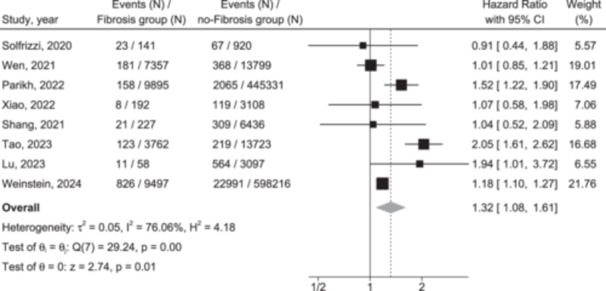
Forest plot depicting the pooled effect size of the association between liver fibrosis and the risk of developing dementia across eight eligible cohort studies.

Figure 2 illustrates the distribution of included studies evaluating the relationship between liver fibrosis and the risk of incident dementia. A meta‐analysis of eight eligible cohorts, comprising aggregate data on 1,115,759 middle‐aged individuals (56.5% of them were women, average age 55.8 years [SD: 8.4]), with 31,129 participants having liver fibrosis at baseline, identified 29,923 cases of dementia over a mean follow‐up period of 13.97 years (SD: 4.02). The presence of liver fibrosis was associated with a moderately increased risk of incident dementia (pooled HR: 1.32, 95% CI: 1.08–1.61, *p* = 0.006; *I*² = 76.06%).

Since fully adjusted HR were consistently extracted and pooled, the pooled HR remained independent of age, sex, alcohol consumption, measures of adiposity, smoking status, diabetes, hypertension, dyslipidemia, and other prevalent cardiometabolic risk factors. This independence was further validated through a sensitivity analysis restricted to studies with the maximum level of covariate adjustment (*n* = 5), which demonstrated a similar association (pooled HR: 1.29, 95% CI: 1.07–1.56, *I*² = 45.28%).

**Figure 3:**
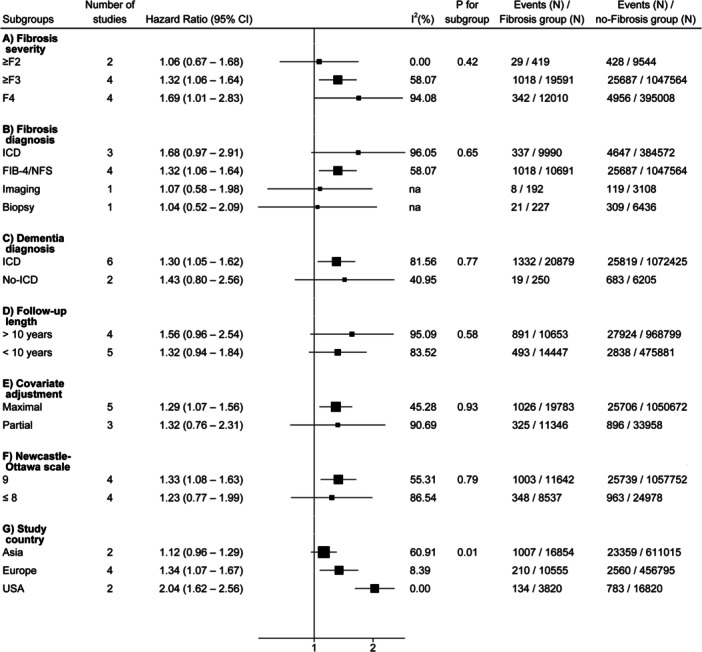
Subgroup analyses were conducted to evaluate the associations between liver fibrosis and the risk of incident dementia, categorized by fibrosis severity, fibrosis diagnosis method, dementia diagnosis method, mean follow‐up duration, level of covariate adjustment, risk of bias (evaluated using the Newcastle–Ottawa Scale), and study location. FIB‐4, Fibrosis‐4 Index; ICD, International Classification of Diseases; NFS, NAFLD fibrosis score; USA, United States of America.

The risk of dementia increased incrementally with the liver fibrosis severity, as shown in Figure 3. The pooled HRs were as follows: significant fibrosis (≥ F2): 1.06 (95% CI: 0.67–1.68, *I*² = 0.00%), advanced fibrosis (≥ F3): 1.32 (95% CI: 1.06–1.64, *I*² = 58.07%), and cirrhosis (F4): 1.69 (95% CI: 1.01–2.83, *I*² = 94.08%).

### 3.4 Subgroup Analysis

Subgroup analyses were conducted to explore potential sources of heterogeneity among the included studies (Figure 3). The link between liver fibrosis and incident dementia remained stable across stratifications based on fibrosis severity, diagnostic methods for fibrosis and dementia, extent of covariate adjustment, risk of bias (NOS), and follow‐up duration. However, regional subgroup analysis revealed significant heterogeneity at the continental level (*p* for subgroup = 0.01). The pooled HRs were 1.12 (95% CI: 0.96–1.29, *I*² = 60.91%) in Asia, 1.34 (95% CI: 1.07–1.67, *I*² = 8.39%) in Europe, and 2.04 (95% CI: 1.62–2.56, *I*² = 0.00%) in the USA. This subgroup analysis substantially explained the overall heterogeneity across the included cohorts; however, significant heterogeneity persisted in the Asian subgroup. This may be attributed to the inclusion of one study in Asia that was not primarily designed to assess the relationship between liver fibrosis and incident dementia, instead examining liver fibrosis univariately as a covariate and focusing on Alzheimer's disease rather than all‐cause dementia as the outcome.

### 3.5 Sensitivity Analysis and Publication Bias

To further validate our findings, we conducted several sensitivity analyses. First, we restricted the analysis to studies with the maximum level of covariate adjustment (*n *= 5 studies), yielding a pooled HR of 1.29 (95% CI: 1.07–1.56, *I*² = 45.28%). Second, we included only studies with the lowest risk of bias (NOS = 9), which further supported our results (*n *= 4 studies, pooled HR: 1.33, 95% CI: 1.08–1.63, *I*² = 55.31%). Additionally, a leave‐one‐out analysis demonstrated that excluding any single study had minimal impact on the overall pooled hazard ratio and its corresponding 95% CI (Supporting Information S1: Figure S2), further confirming the robustness and reliability of our findings.

### 3.6 Sex‐ and Cause‐Specific Association of Liver Fibrosis and Dementia

The risk of incident dementia appears higher in women with liver fibrosis (*n* = 2 studies, pooled HR: 1.94, 95% CI: 0.75–5.03, *I*² = 0.00%) compared to men with liver fibrosis (*n* = 2 studies, pooled HR: 1.18, 95% CI: 1.07–1.30, *I*² = 0.00%). However, given the small number of studies eligible for meta‐analysis, these results should be considered within the context of a systematic review rather than a meta‐analysis. Notably, only two cohort studies (Weinstein et al. [11] from Israel and Yuan et al. [27] from the United Kingdom) investigated the relationship between liver fibrosis and incident dementia with sex‐specific manner. While both studies reported a significant association in their individual analyses (Table 1), the pooled HR in women was nonsignificant due to the lack of overlapping confidence intervals and substantial variability between the studies, leading to a wide confidence interval.

Furthermore, liver fibrosis appears to increase the risk of vascular dementia (VD) more than Alzheimer's disease (AD), although cause‐specific analysis can only be discussed systematically. Among the available studies, only those from the UK Biobank analyzed vascular dementia specifically. Parikh et al. [8] reported a significant association with advanced fibrosis (≥ F3, HR: 1.64, 95% CI: 1.03–2.61), while Yuan et al. [27] reported an even higher association with cirrhosis (F4, HR: 3.30, 95% CI: 1.55–3.13). For AD, studies from the UK Biobank, Taiwan, and Sweden cohorts also assessed this outcome. Parikh et al. [8] and Shang et al. [15] examined the risk of AD in patients with significant or advanced fibrosis (≥ F2) and found no significant association (≥ F2, *n* = 2 studies, pooled HR: 1.25, 95% CI: 0.88–1.78, *I*² = 0.00%). Shang et al. [15] also examined AD in patients with cirrhosis (F4), and together with Wen et al. [25] and Yuan et al. [27], the pooled HR for AD remained nonsignificant (F4, *n* = 3 studies, pooled HR: 1.44, 95% CI: 0.82–2.53, *I*² = 86.49%).

## 4 Discussion

### 4.1 Main Findings

In this meta‐analysis of 8 diverse cohorts, including 1,115,759 middle‐aged individuals (56% were women with an average age of 55 years), we identified 31,129 participants with liver fibrosis at baseline and 29,923 new cases of dementia over an average follow‐up of 14 years. Our review led to four novel findings. Firstly, liver fibrosis is associated with a modest though significantly increased risk of developing all‐cause dementia over a long‐term follow‐up (pooled HR: 1.32, 95% CI: 1.08–1.61, *I*² = 76.06%). It is important to pinpoint that the observed association between liver fibrosis and dementia risk is independent of confounding factors, including age, sex, race, ethnicity, education level, measurement of adiposity, smoking habit, alcohol consumption, type 2 diabetes, hypertension, dyslipidemia, and other cardiometabolic risk factors. Sensitivity analyses confirmed the robustness of the findings, with the association persisting after restricting the analysis to studies with low risk of bias (NOS 9) and studies with maximal covariate adjustment. Secondly, the risk of dementia increases incrementally with the severity of liver fibrosis, indicating a dose–response relationship (Figure 3). The pooled HR for significant fibrosis (≥ F2), advanced fibrosis (≥ F3), and cirrhosis (F4) were 1.06 (95% CI: 0.67–1.68), 1.32 (95% CI: 1.06–1.64), and 1.69 (95% CI: 1.01–2.83), respectively. This finding is consistent with previous research showing that the burden of extrahepatic complications, such as cardiovascular disease, tends to rise in parallel with the increasing severity of liver fibrosis [28]. The observed gradient further supports the biological plausibility of a causal relationship, reinforcing the need for early identification and management of liver fibrosis to potentially mitigate downstream neurological outcomes.

Additionally, subgroup analyses revealed notable regional variations at the continental level, with the strongest associations observed in studies conducted in the USA, followed by Europe and Asia (Figure 3). These differences highlight the potential influence of population characteristics, healthcare practices, or environmental factors on the association of liver fibrosis and dementia. Finally, sex‐ and cause‐specific analyses showed that women with fibrosing CLD were at higher risk of incident dementia than men. This finding is supported by accumulating evidence showing that women with CLD, particularly postmenopausal women, are at greater risk of fibrosis progression [29], which may lead to a higher burden of extrahepatic complications, including cerebrovascular events and stroke [9, 30]. These sex‐specific findings highlight the need for precision medicine approaches in liver fibrosis and dementia research and clinical care. Moreover, our data suggest that liver fibrosis is more strongly associated with the risk of VD than AD, likely due to shared metabolic and vascular mechanisms. This is consistent with a recent Mendelian randomization study that found a potential causal relationship between metabolic dysfunction‐associated steatotic liver disease (MASLD)‐related fibrosis and VD, but not AD [31].

### 4.2 Potential Pathomechanisms Involved

Emerging evidence suggests that liver fibrosis may contribute to cognitive decline through multiple interconnected mechanisms, beyond the well‐established hepatic encephalopathy seen in advanced liver disease [26, 32]. Fibrosing CLD is increasingly recognized as a state of persistent low‐grade inflammation, marked by elevated cytokines and immune mediators that impair vascular homeostasis [33]. This subclinical inflammation promotes endothelial dysfunction, small vessel disease, and disruption of the blood–brain barrier, all of which are central to the development of vascular dementia [34]. Additionally, immune activation and oxidative stress further compromise cerebrovascular health [35]. Liver fibrosis is also associated with subclinical atherosclerosis [36] and increased stroke severity [37], which may further contribute to cognitive impairment via impaired cerebral perfusion.

Gut dysbiosis and increased intestinal permeability, common in MASLD and CLD owing to other etiologies, may exacerbate neuroinflammation through the translocation of bacterial endotoxins into the systemic circulation [38, 39, 40, 41]. These gut‐liver‐brain interactions are increasingly recognized as strong contributors to either vascular or neurodegenerative processes [42]. Moreover, CLD‐associated impaired hepatic clearance may lead to intracerebral deposition and accumulation of circulating amyloid‐β—a hallmark of Alzheimer's disease [43, 44]. Supporting this notion, neuroimaging studies have shown reduced volumes of cortical gray matter in patients with MASLD and liver fibrosis [45].

These liver‐related changes in the size of the cerebral cortex may also serve as a sex‐specific mediator of dementia risk in MASLD. Women generally have lower brain volumes than men, a difference that increases with age [46]. Additionally, the postmenopausal vulnerability to liver fibrosis progression [29] may further accelerate brain atrophy and the risk of dementia in women [47]. Moreover, women with liver fibrosis face a higher stroke risk [48], and post‐stroke brain atrophy can further amplify the susceptibility to develop dementia [49]. This sex‐specific liver–brain axis is supported by our pooled analysis, which showed a stronger association between liver fibrosis and dementia in women. However, the potential role of brain volume as a sex‐specific mediator warrants further investigation to quantify its contribution and clarify its significance within the liver–brain axis. Additionally, severe reduction and failure of hepatic detoxification capacity, often associated with portal hypertension, via raised concentrations of circulating neurotoxins, among which ammonemia is the best characterized, may further contribute to cognitive dysfunction even in the absence of overt hepatic encephalopathy [13].

Metabolic dysregulation—featuring insulin resistance, type 2 diabetes, arterial hypertension, and dyslipidemia—is highly prevalent in MASLD and contributes to both liver fibrosis and cognitive decline [50, 51]. These comorbidities may amplify systemic inflammation and vascular injury, thereby accelerating neurodegenerative changes [50]. In this regard, metabolic syndrome has been independently associated with brain structural abnormalities, such as hippocampal atrophy and cortical thinning, even in the absence of overt liver disease [52]. Therefore, metabolic dysfunction probably plays the dual role of shared risk factor and mediator in the liver–brain axis.

Alcohol (ab)use may further modulate the liver‐brain cross‐talks. As a known hepatotoxin, alcohol promotes steatosis, inflammation, and fibrosis [53]. However, alcohol is also directly neurotoxic, and chronic ethanol consumption has been linked to cerebral atrophy, white matter injury, and global cognitive decline [54]. Of concern, alcohol may synergize with metabolic comorbidities in exacerbating both hepatic and neurologic injury [55]. Therefore, given that alcohol consumption represents both a confounder and an effect modifier in this context, future studies should carefully assess pattern, quantity, type, and duration of drinking when evaluating the independent role of liver fibrosis in dementia risk.

### 4.3 Strengths and Limitations

Our meta‐analysis has notable strengths. First, to the best of our knowledge, this is the first systematic review with meta‐analysis documenting the association of liver fibrosis with incident dementia. Second, the substantial sample size (~ 1.1 million) and the large number of incident dementia cases (~ 30,000) that occurred over the mean follow‐up of 14 years provide high statistical power. Additionally, the overall quality of the included studies was acceptable, indicating a medium‐to‐low risk of bias, as reflected by a weighted mean NOS score of 8.3. Moreover, Sensitivity analyses restricted to studies with maximal covariate adjustment and low risk of bias (NOS = 9) further validate the findings. Iterative exclusion of each study from the meta‐analysis also showed that the findings were not disproportionately dependent on any individual study. Finally, no evidence of publication bias was detected based on Egger's regression test and visual inspection of the funnel plot.

Nonetheless, several limitations should be noted. First, the observational nature of the included studies precludes any strong conclusions regarding causality. Second, the primary analysis showed significant heterogeneity, which dictates cautious interpretation. This heterogeneity likely reflects variations among various geographic areas, methods used for capturing liver fibrosis and dementia, gauging fibrosis severity, duration of follow‐up, study quality, and the degree of covariate adjustment. Additionally, although most included studies adjusted their findings for relevant confounding factors, residual confounding from unmeasured factors and reverse association cannot be ruled out with certainty. Therefore, Mendelian randomization studies are warranted to prove cause‐and‐effect relationships. Finally, the power of sex‐ and cause‐specific analyses was limited by the number of available studies and should be interpreted with caution.

## 5 Conclusion

Given disease complex causes and limited treatment options, the prevention of dementia requires a clear understanding of its modifiable risk factors. Our meta‐analysis supports the role of liver fibrosis as a true barometer of systemic health [10] and reveals a modest but statistically significant association with incident dementia over a long‐term follow‐up. These findings underscore the importance of additional well‐designed and well‐conducted studies to clarify the subtype‐specific and sex‐specific risks of dementia, particularly Alzheimer's disease and vascular dementia. Based on our findings, healthcare professionals should recognize the moderately increased risk of developing dementia in individuals with liver fibrosis and perform close surveillance of these patients to enable early detection and timely intervention.

## Author Contributions

**Mohamad Jamalinia:** conceptualization, methodology, data curation, formal analysis, writing – original draft, writing – review and editing. **Fatemeh Zare:** writing – review and editing, data curation. **Amedeo Lonardo:** supervision, writing – original draft, writing – review and editing.

## Conflicts of Interest

The authors declare no conflicts of interest.

## Transparency Statement

The lead author, Mohamad Jamalinia, affirms that this manuscript is an honest, accurate, and transparent account of the study being reported; that no important aspects of the study have been omitted; and that any discrepancies from the study as planned (and, if relevant, registered) have been explained.

## Supporting information

Supporting materials.

## Data Availability

The data that support the findings of this study are available from the corresponding author upon reasonable request. The data used in this article are available in the full text or the supporting material. For further inquiries, readers may contact the corresponding author.
