# Supplementary material for: Liver Fibrosis and Risk of Incident Dementia in the General Population: Systematic Review With Meta‐Analysis
Source: Health Sci Rep. 2025 Nov 17;8(11):e71530. doi: 10.1002/hsr2.71530 (PMC12623456; doi:10.1002/hsr2.71530)
Supplement: Supplementary file 1 — Supporting materials. [file HSR2-8-e71530-s001.pdf]

Supplementary materials

Mohamad Jamalinia, Fatemeh Zare, Amedeo Lonardo

Table S1. Keywords used through database searching on PubMed, Embase, Scopus, or Web of Science

Table S2. Syntax used through database searching on PubMed, Embase, Scopus, or Web of Science

Figure S1. Funnel plot of standard error by log-hazard ratio for the risk of incident dementia (for the studies included in Figure 2).

Figure S2. Leave-one-out analysis showing the effect of omitting individual studies (for the 8 studies included in Figure 2). The effect size is expressed as random-effects hazard ratio (HR) and 95% confidence intervals (CIs).

Figure S3. Risk of bias summary for each eligible study, assessed using the Cochrane Collaboration's tool.

Figure S4. Risk of bias graph summarizing the overall assessment of all eligible studies, based on the Cochrane Collaboration's tool.

Table S3. Newcastle-Ottawa Quality Assessment Scale (NOS) scores for the studies included in the meta-analysis.

Table S1. Keywords used through database searching on PubMed, Embase, Scopus, or Web of Science

	Exposure	Outcome
**Keywords**	(("liver" ***OR*** "hepatic") ***AND*** ("biops*" ***OR*** "fibros*" ***OR*** "cirrhos*" ***OR*** "stiffness*" ***OR*** "puncture" ***OR*** "elastogra*" ***OR*** "elasticit*" ***OR*** "acoustography" ***OR*** "vibroacoustography" ***OR*** "vibro-acoustography" ***OR*** "sonoelastograph*" ***OR*** "fibroscan" ***OR*** "acoustic radiation force impulse imaging" ***OR*** "arfi imaging*")	"Dementia*" ***OR*** "Alzheimer*" ***OR*** "Binswanger encephalopathy" ***OR*** "CADASIL" ***OR*** "Lewy body disease" ***OR*** "Neurofibrillary tangles with calcification" ***OR*** "Primary progressive aphasia" ***OR*** "Progressive nonfluent aphasia" ***OR*** "Hereditary diffuse leukoencephalopathy with spheroids" ***OR*** "Huntington chorea" ***OR*** "Kluver-Bucy syndrome" ***OR*** "Mental deterioration" ***OR*** "Nasu-Hakola disease" ***OR*** "Neuronal ceroid lipofuscinosis" ***OR*** "Prion disease" ***OR*** "Bovine spongiform encephalopathy" ***OR*** "Chronic wasting disease" ***OR*** "Creutzfeldt-Jakob disease" ***OR*** "Feline spongiform encephalopathy" ***OR*** "Fatal familial insomnia" ***OR*** "Gerstmann-Straussler-Scheinker syndrome" ***OR*** "Kuru" ***OR*** "Scrapie" ***OR*** "Transmissible mink encephalopathy" ***OR*** "Variably protease-sensitive prionopathy" ***OR*** "Pseudodementia" ***OR*** "Rett syndrome" ***OR*** "Senility" ***OR*** "Tauopathy" ***OR*** "Creutzfeldt-Jakob syndrome" ***OR*** "Diffuse neurofibrillary tangles with calcification" ***OR*** "Frontotemporal lobar degeneration" ***OR*** "Huntington disease" ***OR*** "Amentia*"
**Mesh**	"liver" ***AND*** ("Elasticity Imaging Techniques" ***OR*** "biopsy" ***OR*** "fibrosis")	"Dementia"
**Emtree**	"liver fibrosis" ***OR*** ("liver" ***AND*** ("elastograph" ***OR*** "elastography")) ***OR*** "liver biopsy"	"Dementia"

**Table S2**. Syntax used through database searching on PubMed, Embase, Scopus, or Web of Science		
**Database**	**Syntax**	**Results**
**PubMed**	(("liver"[Mesh] AND ("Elasticity Imaging Techniques"[Mesh] ***OR*** "biopsy"[Mesh] ***OR*** "fibrosis"[Mesh])) ***OR*** "Liver Cirrhosis"[Mesh] ***OR*** (("liver"[tiab] ***OR*** "hepatic"[tiab]) AND ("biops*"[tiab] ***OR*** "fibros*"[tiab] ***OR*** "cirrhos*"[tiab] ***OR*** "stiffness*"[tiab] ***OR*** "puncture"[tiab] ***OR*** "elastogra*"[tiab] ***OR*** "elasticit*"[tiab] ***OR*** "acoustography"[tiab] ***OR*** "vibroacoustography"[tiab] ***OR*** "vibro-acoustography"[tiab] ***OR*** "sonoelastograph*"[tiab] ***OR*** "fibroscan"[tiab] ***OR*** "acoustic radiation force impulse imaging"[tiab] ***OR*** "arfi imaging*"[tiab]))) ***AND*** ("Dementia"[Mesh] ***OR*** "Dementia*"[tiab] ***OR*** "Alzheimer*"[tiab] ***OR*** "Binswanger encephalopathy"[tiab] ***OR*** "CADASIL"[tiab] ***OR*** "Lewy body disease"[tiab] ***OR*** "Neurofibrillary tangles with calcification"[tiab] ***OR*** "Primary progressive aphasia"[tiab] ***OR*** "Progressive nonfluent aphasia"[tiab] ***OR*** "Hereditary diffuse leukoencephalopathy with spheroids"[tiab] ***OR*** "Huntington chorea"[tiab] ***OR*** "Kluver-Bucy syndrome"[tiab] ***OR*** "Mental deterioration"[tiab] ***OR*** "Nasu-Hakola disease"[tiab] ***OR*** "Neuronal ceroid lipofuscinosis"[tiab] ***OR*** "Prion disease"[tiab] ***OR*** "Bovine spongiform encephalopathy"[tiab] ***OR*** "Chronic wasting disease"[tiab] ***OR*** "Creutzfeldt-Jakob disease"[tiab] ***OR*** "Feline spongiform encephalopathy"[tiab] ***OR*** "Fatal familial insomnia"[tiab] ***OR*** "Gerstmann-Straussler-Scheinker syndrome"[tiab] ***OR*** "Kuru"[tiab] ***OR*** "Scrapie"[tiab] ***OR*** "Transmissible mink encephalopathy"[tiab] ***OR*** "Variably protease-sensitive prionopathy"[tiab] ***OR*** "Pseudodementia"[tiab] ***OR*** "Rett syndrome"[tiab] ***OR*** "Senility"[tiab] ***OR*** "Tauopathy"[tiab] ***OR*** "Creutzfeldt-Jakob syndrome"[tiab] ***OR*** "Diffuse neurofibrillary tangles with calcification"[tiab] ***OR*** "Frontotemporal lobar degeneration"[tiab] ***OR*** "Huntington disease"[tiab] ***OR*** "Amentia*"[tiab]) ***AND*** ("1950/01/01"[Date - Publication] : "2024/10/22"[Date - Publication])	**493**
**Embase**	("liver fibrosis"/de ***OR*** ("liver"/de AND ("elastograph"/de ***OR*** "elastography"/de)) ***OR*** "liver biopsy"/de ***OR*** (("liver":ab,ti ***OR*** "hepatic":ab,ti) AND ("biops*":ab,ti ***OR*** "fibros*":ab,ti ***OR*** "cirrhos*":ab,ti ***OR*** "stiffness*":ab,ti ***OR*** "puncture":ab,ti ***OR*** "elastogra*":ab,ti ***OR*** "elasticit*":ab,ti ***OR*** "acoustography":ab,ti ***OR*** "vibroacoustography":ab,ti ***OR*** "vibro-acoustography":ab,ti ***OR*** "sonoelastograph*":ab,ti ***OR*** "fibroscan":ab,ti ***OR*** "acoustic radiation force impulse imaging":ab,ti ***OR*** "arfi imaging*":ab,ti))) **AND** ("Dementia"/de ***OR*** "Dementia*":ab,ti ***OR*** "Alzheimer*":ab,ti ***OR*** "Binswanger encephalopathy":ab,ti ***OR*** "CADASIL":ab,ti ***OR*** "Lewy body disease":ab,ti ***OR*** "Neurofibrillary tangles with calcification":ab,ti ***OR*** "Primary progressive aphasia":ab,ti ***OR*** "Progressive nonfluent aphasia":ab,ti ***OR*** "Hereditary diffuse leukoencephalopathy with spheroids":ab,ti ***OR*** "Huntington chorea":ab,ti ***OR*** "Kluver-Bucy syndrome":ab,ti ***OR*** "Mental deterioration":ab,ti ***OR*** "Nasu-Hakola disease":ab,ti ***OR*** "Neuronal ceroid lipofuscinosis":ab,ti ***OR*** "Prion disease":ab,ti ***OR*** "Bovine spongiform encephalopathy":ab,ti ***OR*** "Chronic wasting disease":ab,ti ***OR*** "Creutzfeldt-Jakob disease":ab,ti ***OR*** "Feline spongiform encephalopathy":ab,ti ***OR*** "Fatal familial insomnia":ab,ti ***OR*** "Gerstmann-Straussler-Scheinker	**450**

	syndrome":ab,ti ***OR*** "Kuru":ab,ti ***OR*** "Scrapie":ab,ti ***OR*** "Transmissible mink encephalopathy":ab,ti ***OR*** "Variably protease-sensitive prionopathy":ab,ti ***OR*** "Pseudodementia":ab,ti ***OR*** "Rett syndrome":ab,ti ***OR*** "Senility":ab,ti ***OR*** "Tauopathy":ab,ti ***OR*** "Creutzfeldt-Jakob syndrome":ab,ti ***OR*** "Diffuse neurofibrillary tangles with calcification":ab,ti ***OR*** "Frontotemporal lobar degeneration":ab,ti ***OR*** "Huntington disease":ab,ti ***OR*** "Amentia*":ab,ti) NOT [medline]/lim ***AND*** [01-01-1950]/sd NOT [23-10-2024]/sd	
**Scopus**	TITLE-ABS-KEY(("liver" ***OR*** "hepatic") AND ("biops*" ***OR*** "fibros*" ***OR*** "cirrhos*" ***OR*** "stiffness*" ***OR*** "puncture" ***OR*** "elastogra*" ***OR*** "elasticit*" ***OR*** "acoustography" ***OR*** "vibroacoustography" ***OR*** "vibro-acoustography" ***OR*** "sonoelastograph*" ***OR*** "fibroscan" ***OR*** "acoustic radiation f***OR***ce impulse imaging" ***OR*** "arfi imaging*")) ***AND*** TITLE-ABS-KEY ("Dementia*" ***OR*** "Alzheimer*" ***OR*** "Binswanger encephalopathy" ***OR*** "CADASIL" ***OR*** "Lewy body disease" ***OR*** "Neurofibrillary tangles with calcification" ***OR*** "Primary progressive aphasia" ***OR*** "Progressive nonfluent aphasia" ***OR*** "Hereditary diffuse leukoencephalopathy with spheroids" ***OR*** "Huntington chorea" ***OR*** "Kluver-Bucy syndrome" ***OR*** "Mental deterioration" ***OR*** "Nasu-Hakola disease" ***OR*** "Neuronal ceroid lipofuscinosis" ***OR*** "Prion disease" ***OR*** "Bovine spongiform encephalopathy" ***OR*** "Chronic wasting disease" ***OR*** "Creutzfeldt-Jakob disease" ***OR*** "Feline spongiform encephalopathy" ***OR*** "Fatal familial insomnia" ***OR*** "Gerstmann-Straussler-Scheinker syndrome" ***OR*** "Kuru" ***OR*** "Scrapie" ***OR*** "Transmissible mink encephalopathy" ***OR*** "Variably protease-sensitive prionopathy" ***OR*** "Pseudodementia" ***OR*** "Rett syndrome" ***OR*** "Senility" ***OR*** "Tauopathy" ***OR*** "Creutzfeldt-Jakob syndrome" ***OR*** "Diffuse neurofibrillary tangles with calcification" ***OR*** "Frontotemporal lobar degeneration" ***OR*** "Huntington disease" ***OR*** "Amentia*")	**2404**
**Web of Science**	TS=(("liver" ***OR*** "hepatic") AND ("biops*" ***OR*** "fibros*" ***OR*** "cirrhos*" ***OR*** "stiffness*" ***OR*** "puncture" ***OR*** "elastogra*" ***OR*** "elasticit*" ***OR*** "acoustography" ***OR*** "vibroacoustography" ***OR*** "vibro-acoustography" ***OR*** "sonoelastograph*" ***OR*** "fibroscan" ***OR*** "acoustic radiation force impulse imaging" ***OR*** "arfi imaging*")) ***AND*** TS=("Dementia*" ***OR*** "Alzheimer*" ***OR*** "Binswanger encephalopathy" ***OR*** "CADASIL" ***OR*** "Lewy body disease" ***OR*** "Neurofibrillary tangles with calcification" ***OR*** "Primary progressive aphasia" ***OR*** "Progressive nonfluent aphasia" ***OR*** "Hereditary diffuse leukoencephalopathy with spheroids" ***OR*** "Huntington chorea" ***OR*** "Kluver-Bucy syndrome" ***OR*** "Mental deterioration" ***OR*** "Nasu-Hakola disease" ***OR*** "Neuronal ceroid lipofuscinosis" ***OR*** "Prion disease" ***OR*** "Bovine spongiform encephalopathy" ***OR*** "Chronic wasting disease" ***OR*** "Creutzfeldt-Jakob disease" ***OR*** "Feline spongiform encephalopathy" ***OR*** "Fatal familial insomnia" ***OR*** "Gerstmann-Straussler-Scheinker syndrome" ***OR*** "Kuru" ***OR*** "Scrapie" ***OR*** "Transmissible mink encephalopathy" ***OR*** "Variably protease-sensitive prionopathy" ***OR*** "Pseudodementia" ***OR*** "Rett syndrome" ***OR*** "Senility" ***OR*** "Tauopathy" ***OR*** "Creutzfeldt-Jakob syndrome" ***OR*** "Diffuse neurofibrillary tangles with calcification" ***OR*** "Frontotemporal lobar degeneration" ***OR*** "Huntington disease" ***OR*** "Amentia*") ***AND*** DOP=(1950-01-01/2024-10-22)	**563**

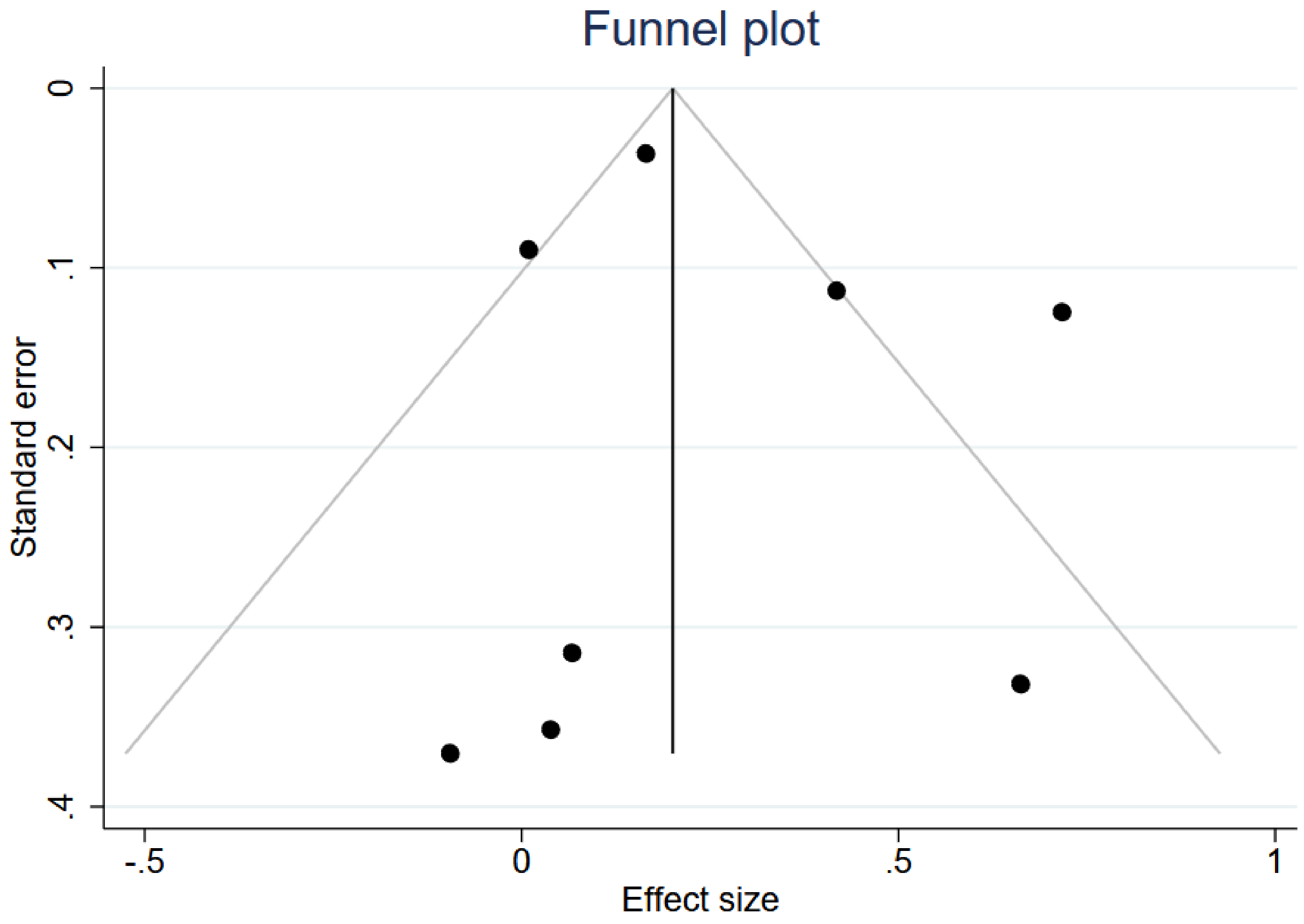

Figure S1. Funnel plot of standard error by log-hazard ratio for the risk of incident dementia (for the studies included in Figure 2).

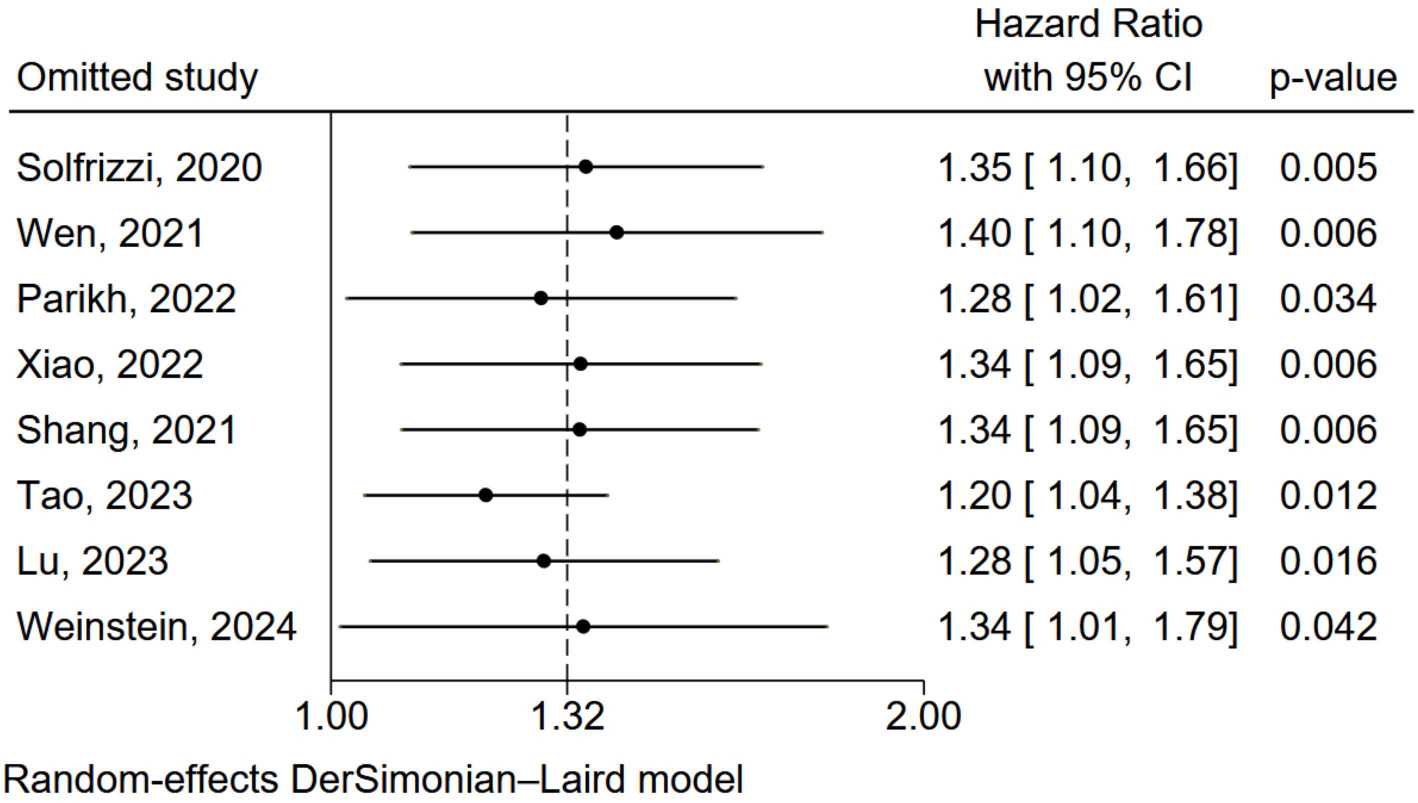

Figure S2. Leave-one-out analysis showing the effect of omitting individual studies (for the 8 studies included in Figure 2). The effect size is expressed as random-effects hazard ratio (HR) and 95% confidence intervals (CIs).

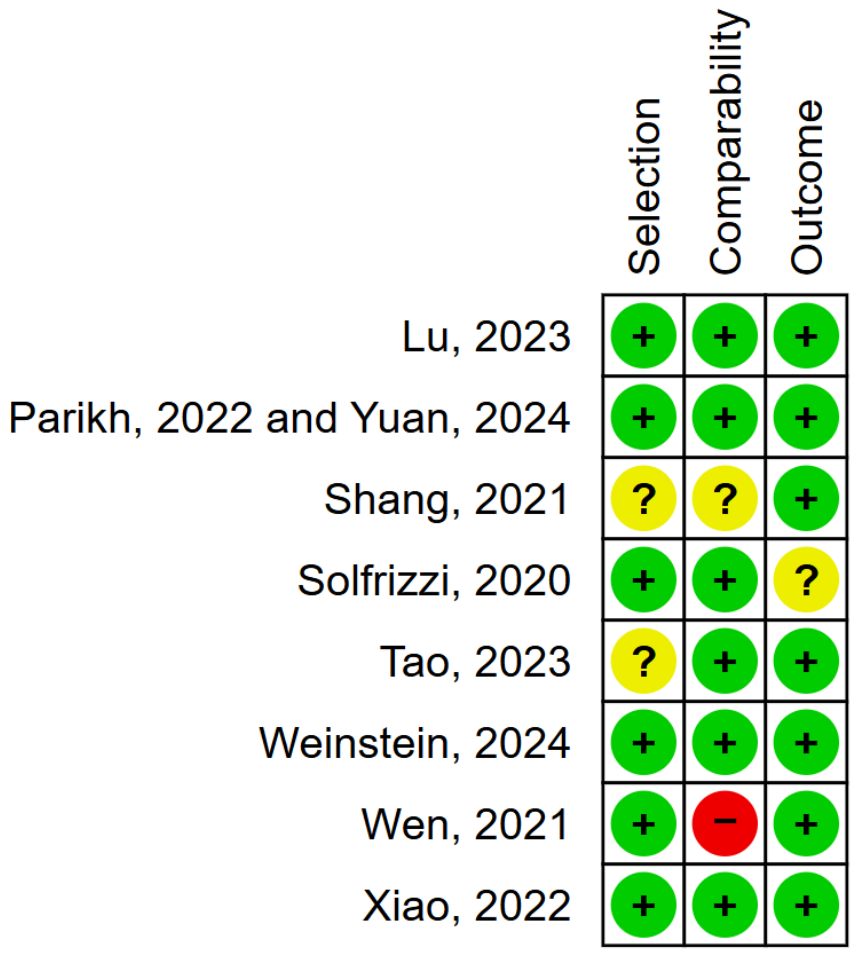

Figure S3. Risk of bias summary for each eligible study, assessed using the Cochrane Collaboration's tool.

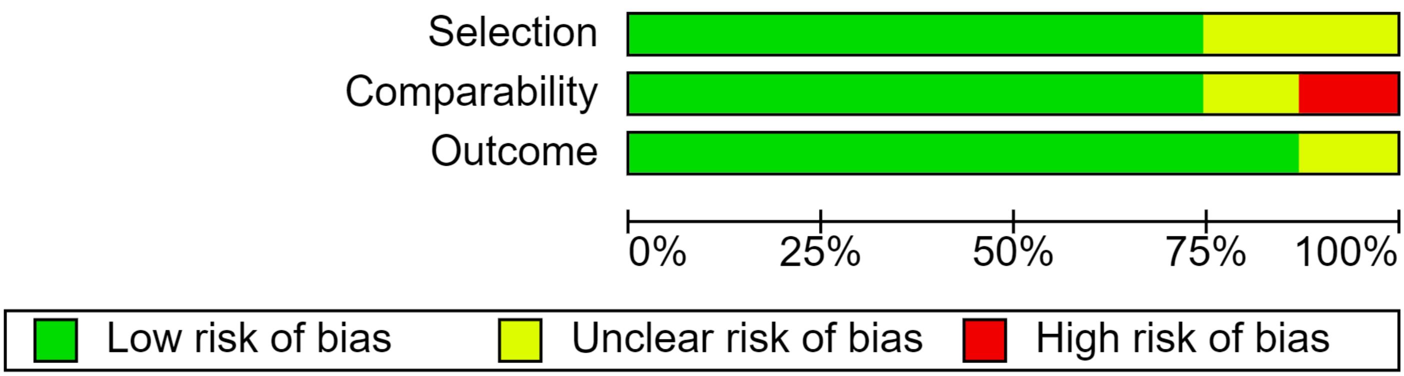

Figure S4. Risk of bias graph summarizing the overall assessment of all eligible studies, based on the Cochrane Collaboration's tool.

Table S3. Newcastle-Ottawa Quality Assessment Scale (NOS) for the studies included in the meta-analysis.

Study	Selection	Comparability	Outcome
Solfrizzi et al., 2020	****	**	**
Wen et al., 2021	****		***
Shang et al., 2021	***	*	***
Parikh et al., 2022 And Yuan et al., 2024	****	**	***
Xiao et al., 2022	****	**	***
Tao et al., 2023	***	**	***
Lu et al., 2023	****	**	***
Weinstein et al., 2024	****	**	***
